# Comparison and evaluation of pair distribution functions, using a similarity measure based on cross-correlation functions

**DOI:** 10.1107/S1600576721001722

**Published:** 2021-03-31

**Authors:** Stefan Habermehl, Carina Schlesinger, Dragica Prill

**Affiliations:** a Institute of Inorganic and Analytical Chemistry, Goethe University, Max-von-Laue-Strasse 7, 60438 Frankfurt am Main, Germany

**Keywords:** pair distribution functions, similarity measures, total scattering techniques, cross-correlation functions, *R* values

## Abstract

An approach for the comparison of pair distribution functions (PDFs) has been developed using a similarity measure based on cross-correlation functions. The PDF is very sensitive to changes in the local structure, *i.e.* small deviations in the structure can cause large signal shifts and significant discrepancies between the PDFs. Therefore, a comparison based on pointwise differences (*e.g. R* values and difference curves) may lead to the assumption that the investigated PDFs as well as the corresponding structural models are not in agreement at all, whereas a careful visual inspection of the investigated structural models and corresponding PDFs may reveal a relatively good match. To quantify the agreement of different PDFs for those cases an alternative approach is introduced: the similarity measure based on cross-correlation functions. In this paper, the power of this application of the similarity measure to the analysis of PDFs is highlighted. The similarity measure is compared with the classical *R*_wp_ values as representative of the comparison based on pointwise differences as well as with the Pearson product-moment correlation coefficient, using polymorph IV of barbituric acid as an example.

## Abstract 2

A novel approach to the quantification of the agreement between pair distribution functions by a similarity measure based on cross-correlation functions is introduced and evaluated.

## 1. Introduction   

The analysis of the atomic pair distribution function (PDF) is the method of choice to investigate amorphous or nanocrystalline samples, as frequently found in nature (Poulain *et al.*, 2019 ▸) and in novel, complex, engineered materials (Cliffe *et al.*, 2010 ▸; Young & Goodwin, 2011 ▸; Zobel *et al.*, 2015 ▸; Roeser *et al.*, 2017 ▸; Usher *et al.*, 2018 ▸; Billinge, 2019 ▸), *e.g.* metal–organic frameworks (Bennett & Cheetham, 2014 ▸; Mazaj *et al.*, 2016 ▸; Castillo-Blas *et al.*, 2020 ▸), glasses and pharmaceuticals (Moore *et al.*, 2009 ▸; Nollenberger *et al.*, 2009 ▸; Thakral *et al.*, 2016 ▸; Shi *et al.*, 2017 ▸).

The structure of materials can be described by radial distribution functions that give the probability of finding pairs of atoms separated by a distance of *r* as a function of *r*. Information about this probability distribution can be obtained by various experimental methods, *e.g.* X-ray absorption spectroscopy (EXAFS, XANES) or various diffraction methods (X-ray, neutron, electron). The PDF 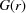 is a specific conceptualization of the approach to the radial distribution on the basis of diffraction measurements (Keen, 2001 ▸). It represents the sum of the contributions of all atom pairs, each weighted by the scattering power of the two atoms of the pair. 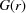 refers to the deviation of the microscopic atomic pair density 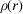 from the average atomic number density 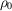 and is described as follows: 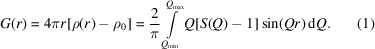


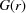
 can be obtained from powder diffraction data by a sine Fourier transformation of the corrected, normalized, diffracted intensity 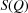 with the magnitude of the scattering vector 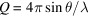, where θ is the scattering angle and λ the wavelength of the radiation used (Egami & Billinge, 2012 ▸). The common analysis of diffraction patterns focusing on the Bragg reflections primarily provides information on the spatial average and long-range order of the investigated material. The analysis of the PDF in contrast is a total scattering technique revealing structural features related to disorder or breakdown of the spatial periodicity.

Crystal structure models imply models of the local or nanoscale structure of a material. Typically a crystallographic unit represents a compact description of the structure and local arrangement of molecules and atoms within a very small volume. Crystal structures, whether determined from measurements or predicted by computational methods, represent thermodynamic minima. The various intra- and intermolecular contributions to the energy balance are clearly dominated by very small distance interactions. By examining the PDFs obtained from the measurements of (nano)crystalline materials or from crystal structure models, the focus is put on the local structure. Moving from single-crystal diffraction to powder diffraction of nanocrystalline materials, it becomes more and more difficult and in the end impossible to extract the structural information from the measurements by analyses that focus on the Bragg reflections and the corresponding long-range periodic order. The shrinking of domain sizes as well as various forms of disorder can be interpreted and represented as increasing perturbations of the ideal order described by a crystal structure model. The local structure of a nanocrystalline material is still dominated by regions of several hundreds of, more or less deviating, unit cells of a structural model while not providing any useful Bragg reflection. Amorphous materials can also exhibit preferred molecular arrangements within a certain small volume that roughly correspond to clusters of unit cells of a structural model. Thus, while on the one hand many good reasons exist for approaching the analysis of the local structure of materials using crystal structure models, on the other hand it is increasingly important to be able to detect and follow rough matches of structural models and experimental data within small spatial ranges.

The PDF can be calculated from a structural model based on a sum over all pairs of atoms *i* and *j* separated by the distance 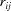: 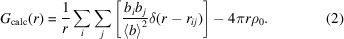
The contribution of each atom–atom pair is represented by a delta function δ at 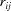 and weighted by the scattering power of the two atoms. The scattering power of atom *i* is 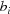 and 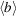 is the average scattering power of the sample. In the instance of neutron scattering 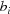 is simply the scattering length and in the case of X-rays it is the atomic form factor evaluated at a user-defined value of *Q*. Alternatively to this real-space approach, the PDF can also be calculated from a structural model via reciprocal space (Neder & Proffen, 2020 ▸).

**Figure 1:**
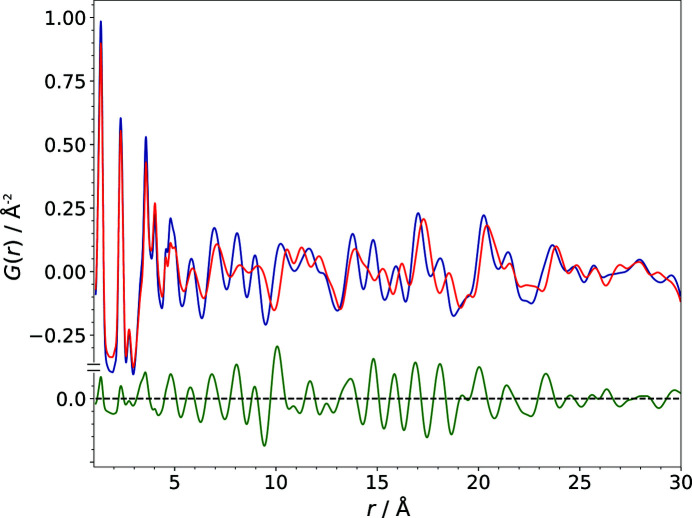
A comparison of the PDFs calculated from the correct structure of polymorph IV of barbituric acid (blue) and a structural model that only differs from the correct one by 0.5 Å in the length of the lattice parameter *b* (red). The difference curve is shown in green.

The PDF is very sensitive to changes in the local structure (Egami & Billinge, 2012 ▸). Hence, comparatively small deviations in the structure can cause significant shifts of signal positions (see Fig. 1 ▸). Correspondingly, the PDF calculated from a crystal structure model is strongly affected by deviations of the lattice parameters, molecular position, molecular orientation or torsion angles.

The evaluation of the similarity or dissimilarity of two PDFs is a common and crucial task in the investigation of the local structures of materials. In particular it is frequently necessary to check if the simulated PDF of a structural model, *e.g.* from a crystal structure prediction or a molecular dynamics simulation, is in considerable agreement with an experimental data set.

The comparison of two PDFs is usually visualized and quantified on the basis of pointwise differences, *i.e.* by a difference curve (see Fig. 1 ▸) and an *R* value, respectively. The agreement of a structural model with an experimental PDF data set is commonly described analogously to residuals such as the weighted-profile *R* value 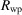 in Rietveld refinements (David, 2004 ▸), *e.g.* with the weighted agreement factor 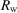 [equation (3)] used in the program *PDFfit* (Egami & Billinge, 2012 ▸): 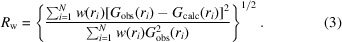


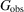
 is the observed value and 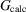 the calculated value at the interatomic distance 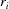, and the corresponding weight for each data point *i* is 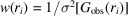 with 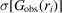 the error of the observed value.


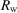
 and similar concepts based on pointwise differences are adequate tools for the comparison and fitting of a structural model to an experimental PDF if the structural model is already very close to the best match. In the case of slightly larger deviations, however, the 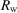 value proves to be an insufficient tool in view of the shifts of the signal positions in the direction of the *r* axis. This is particularly true for deviations of the lattice parameters of a crystal structural model. Visual inspection of the calculated and the observed PDF could still reveal a significant similarity of the two patterns, corresponding to a rough match of the underlying structures, whereas the pointwise comparison of the PDFs results in large discrepancies between the two curves, causing a high 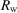 and a difference curve exhibiting large amplitudes.

An alternative approach to PDF comparison uses measures from correlation and regression statistics that are not based on differences, *e.g.* non-parametric approaches such as Spearman’s rank-order correlation and in particular the parametric linear correlation coefficient Pearson’s *r* (Dykhne *et al.*, 2011 ▸; Egami & Billinge, 2012 ▸; Davis *et al.*, 2013 ▸; Shi *et al.*, 2017 ▸; Bordet, 2018 ▸), also known as the product-moment correlation coefficient,
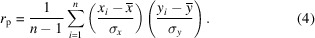


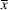
 and 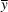 denote the mean values of the two variables *x* and *y*, and 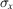 and 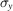 their standard deviations. Here, 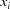 and 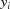 correspond to the values 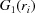 and 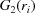 of the two PDFs to be compared. After introduction of the standard deviations in equation (4), Pearson’s *r* can be written as follows: 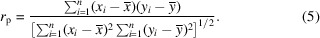


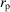
 can take values between −1 for complete anti-correlation and 1 corresponding to complete correlation, with a value of 0 implying no correlation. The measure is also an established tool for the comparison of powder patterns (Gilmore *et al.*, 2004 ▸; Barr *et al.*, 2009 ▸). The Pearson correlation ignores absolute scaling, but is sensitive to slight shifts in peak positions (Dykhne *et al.*, 2011 ▸).

A comparison of the impact of structural changes on the PDF with their impact on powder diagrams elucidates an important difference in that in a powder pattern only variations of the lattice parameters cause signal shifts, while changes of the molecular geometry, position or orientation are reflected in changes of intensities.

The comparison and fitting of structural models to powder data already tend to raise problems if the lattice parameters deviate significantly (Habermehl *et al.*, 2014 ▸). Approaches based on pointwise differences of two curves are very sensitive to shifts in signal positions and tend to fail or become indecisive if the shifts are too large. This problem is even more pronounced in the case of PDFs, because practically every change of the structural model leads to signal shifts in the PDF.

Because of these facts, the comparison of two PDFs is a challenge. This is especially true when a large number of structural models have to be compared, *e.g.* during a structure determination from unindexable powder data by a global fit to the PDF (Schlesinger *et al.*, 2021 ▸).

In this paper, an alternative approach for the quantification of the similarity of two PDFs is reported and applied to an experimental PDF of barbituric acid. The proposed method aims to overcome the shortcomings of the comparison of PDFs based on pointwise differences, using cross-correlation functions in order to detect rough matches of the PDFs that are otherwise obscured by the large discrepancies resulting from the shifts in signal positions. The decisive advantage of the method is that the comparison is based on the product of function values instead of differences, and that the function values within a certain neighbouring range are also taken into account. This is achieved by the use of the generalized similarity measure 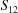 introduced by de Gelder *et al.* (2001 ▸). The approach has already proven to be a valuable tool in the investigation of powder diffraction data. In particular, it has been used very efficiently and successfully as the cost function in the fitting of structural models with strongly deviating lattice parameters to experimental powder data using the program *FIDEL* (Habermehl *et al.*, 2014 ▸, 2021 ▸), even in the instance of ‘problematic’ powder patterns of very low quality.

## 2. Method   

The generalized similarity measure 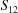 is based on auto- and cross-correlation functions of the two patterns to be compared, *e.g.* an experimental data set and a data set calculated from a structural model. It correlates data points within a given neighbouring range. 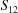 is a very efficient tool for the detection and quantification of rough similarities of two patterns, especially where comparison measures based on pointwise differences cannot adequately reflect the similarity, even in the case of moderate signal shifts or intensity variations.

**Figure 2:**
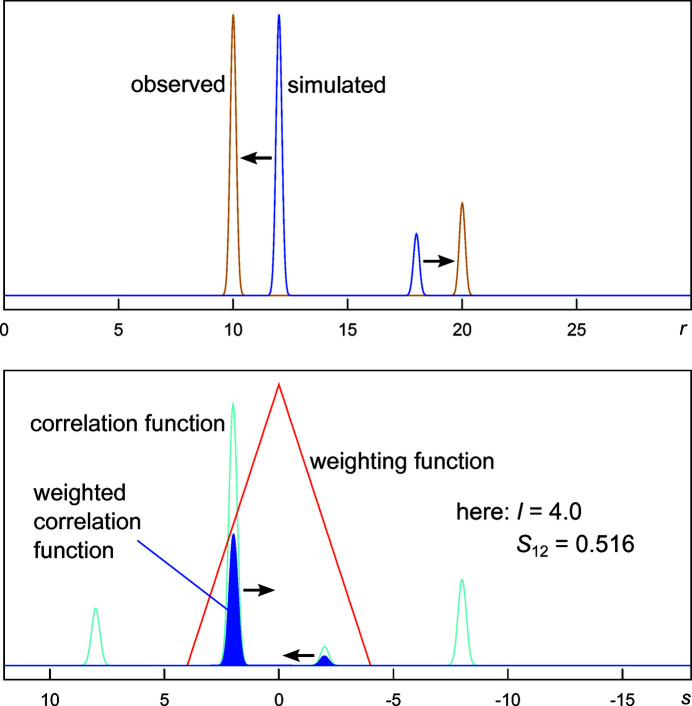
Schematic illustration of the integral (dark-blue area) of the product of the weighting function (red) and the cross-correlation function (light blue) of two simple patterns.

The cross-correlation function 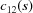 of the two functions 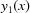 and 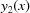 correlates each data point of one curve to the data points at the distance of *s* in the other pattern: 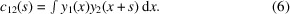
The auto-correlation functions 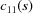 and 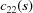 of the two curves are described analogously. The correlation of the data points is restricted to a defined neighbouring range of 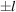 by introducing the triangular weighting function 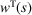: 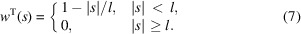
The generalized similarity measure 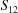 according to de Gelder *et al.* (2001 ▸) is finally obtained by an integration over the weighted correlation function and normalization with respect to the integral values of the weighted auto-correlation functions of the two patterns: 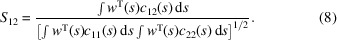


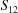
 can take values from 0 to 1, where the value of 1 corresponds to identical patterns. By variation of the half-width *l* of the triangular weighting function, the similarity measure can be adapted to the specific characteristics of the investigated data or problem. Fig. 2 ▸ illustrates the logic behind pattern matching based on the integration over the weighted cross-correlation function of two curves that exhibit a very similar pattern but significant shifts in signal positions. From the simple example depicted in Fig. 2 ▸ it is very clear that a pointwise comparison of the two curves cannot reveal any correlation between them and that the derivatives of an *R* value cannot hint at changes to parameters of a structural model that shift the signals in the correct direction.

This approach of quantifying pattern similarities has been developed and applied in the analysis of measured counts or relative intensities that cannot be negative, such as various kinds of spectra or diffraction measurements. Atomic PDFs, however, are usually described as a difference curve 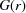 expressing the summed probability of the scattering distributions of atom pairs in relation to the average scattering of the sample [equation (1)]. Hence, 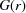 exhibits positive and negative values. Furthermore, the scaling of the PDF is very much dependent on the source of the data such as different measurements or calculation methods. In order to account for the mentioned characteristics of the similarity calculation and the input data, a linear transformation (LT) of the PDFs is performed prior to comparison, such that their baseline is at a value of 1 and their minimum at a value of 0: 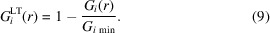
In this simple, stable and robust approach the generalized similarity measure for PDF curves 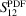 is defined, based on the integrals over the weighted cross-correlation functions, 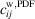 as follows: 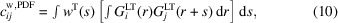


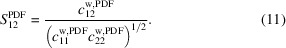
The similarity measure 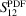 is invariant to the exchange of the two PDFs that are compared. It supports versatile utilization for the comparison of experimental data sets, the comparison of structural models via their simulated PDFs, and the comparison and fitting of structural models to experimental PDF data.

For *l* = 0 the weighting function of equation (7) changes to
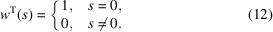
Accordingly, single values of the correlation and auto-correlation functions for *s* = 0 replace the integrals in equation (8). The limit of the similarity measure 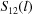 as *l* approaches 0 is denoted here as 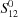: 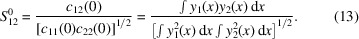
Because PDFs are given not as continuous functions but as sets of discrete data, the similarity measure 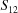 is always calculated using sums in the place of integrals. Accordingly 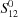 can represent a pointwise comparison of two PDF curves 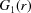 and 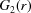 written as
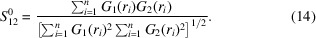
Taking into account that 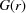 expresses deviations from an average [see equation (1)] and hence the mean 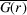 is 0, the comparison of equation (14) with equation (5) shows that 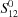 corresponds to Pearson’s *r*.

The limit of 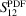 [equation (11)] as *l* approaches 0 is denoted here as 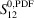. The only difference between 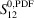 and Pearson’s *r* lies in the use of 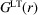 instead of 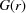. Due to its origin in general statistics the Pearson correlation coefficient is designed to also quantify the possible degree of anti-correlation of two data sets, which is neither reasonable nor desired in the context of its application to the comparison of PDFs. In contrast, 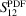 cannot take negative values. In fact, its values are always rather high, which is in line with the physical fact that there is always a basic accordance of the pair distributions and the resulting scattering characteristics of any condensed matter.

## 3. Experimental details   

### 3.1. X-ray powder pattern and experimental PDF   

**Figure 3:**
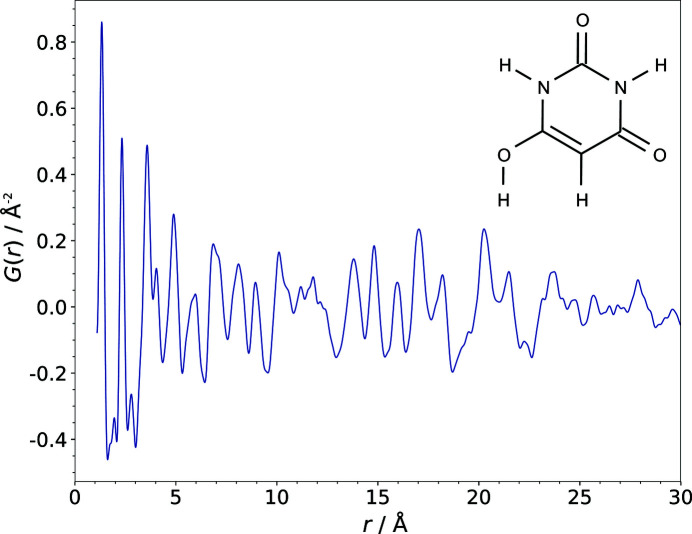
The experimentally obtained PDF of barbituric acid, polymorph IV. The inset shows the structural formula in the enol tautomeric form corresponding to polymorph IV.

Barbituric acid, polymorph II, was purchased from Sigma Aldrich (99% purity) and used without further purification. To obtain polymorph IV of barbituric acid, the sample was milled in a mortar and subsequently placed in a polyimide capillary (1 mm in diameter) which was sealed with clay at both ends. The X-ray powder diagram of the sample was measured at 300 K at the X17A beamline of the National Synchrotron Light Source at Brookhaven National Laboratory. A monochromatic incident X-ray beam conditioned using an Si(311) monochromator to have an energy of 67.42 keV (λ = 0.1839 Å) was used. A 2D PerkinElmer amorphous silicon detector was mounted orthogonally to the beam path with a sample-to-detector distance of 204.2 mm, as calibrated with an LaB_6_ standard sample. Multiple scans of the sample were performed to achieve a total exposure time of 30 min. The 2D diffraction data were integrated and converted to intensity versus 2θ using the software *FIT2D* (Hammersley, 2016 ▸; Hammersley *et al.*, 1996 ▸). The data were corrected, normalized and then truncated at a finite maximum value of the momentum transfer 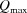, which was optimized to avoid large termination effects whilst maximizing the signal-to-noise ratio, using the program *PDFgetX3* (Juhas *et al.*, 2013 ▸) to obtain the PDF 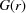. The value 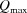 = 21.9 Å^−1^ was found to be optimal for barbituric acid, polymorph IV (Fig. 3 ▸).

### 3.2. Calculation of the PDFs from structural models   

All PDF calculations from structural models were performed with the program *TOPAS-Academic V6* (Coelho *et al.*, 2015 ▸; Coelho, 2018 ▸). In order to calculate the best reliable PDFs from the structural models the calculation with two different isotropic displacement parameters, 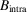 and 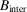 for intra- and intermolecular atom pairs, was used (Prill *et al.*, 2015 ▸). This approach was developed for organic compounds and results in an excellent modelling of both the sharp intramolecular and the broad intermolecular signals in the PDF. An optimal value of the displacement parameter 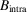 of 0.16 Å^2^ was determined using the calculated PDF of a single barbituric acid molecule. For small organic molecules, a ratio of 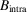 to 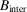 of 1 to 3.75 was observed; hence a value of 0.6 Å^2^ was used for 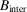 (Prill *et al.*, 2016 ▸). For 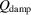, the parameter controlling the instrumental envelope function, a value of 48.0 Å^−1^ was used, based on the measurement of a reference substance. In order to obtain realistic and comparable 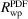 values and difference curves, all PDF calculations were performed using a common scaling of *G*, which was determined by fitting the scale factor of the published structure of barbituric acid, polymorph IV (Schmidt *et al.*, 2011 ▸), to the experimental PDF using *TOPAS*.

### 3.3. Similarity calculations   

The similarity values 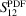 for the comparison of calculated and experimental PDFs were calculated according to the definition given in Section 2 [equation (11)] using the program *FIDEL* (Habermehl *et al.*, 2014 ▸). *FIDEL*’s default value of 0.5 Å for the half-width *l* of the weighting function [equation (7)] was found suitable for the investigations presented here. The similarity of the curves was calculated for the *r* range from 1.1 to 30 Å. The 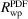 values reported here for comparison are the weighted-pattern *R* values calculated by *TOPAS-Academic V6* (Coelho, 2016 ▸) for the same range and with the static parametrization described in Section 3.2. The Pearson correlation 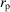 [equation (5)] and the 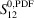 values were also computed by *FIDEL*.

## 4. Application   

### 4.1. Crystal structure of barbituric acid   

**Figure 4:**
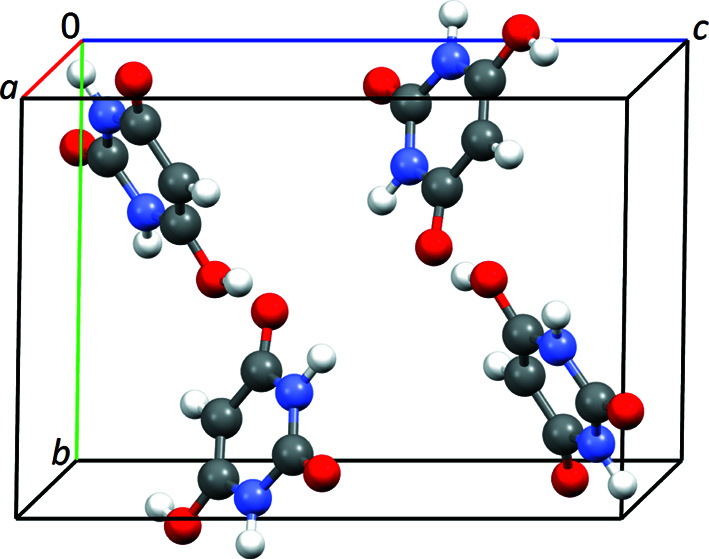
Crystal structure of barbituric acid, polymorph IV, in space group *P*2_1_/*c*.

Barbituric acid [C_4_H_4_N_2_O_3_, pyrimidine-2,4,6(1*H*,3*H*,5*H*)-trione] was chosen as an application example. It forms different polymorphs. At ambient conditions, the thermodynamically stable form is polymorph IV, which contains the enol tautomer. The crystal structure of this polymorph was solved by X-ray and neutron powder data in *P*2_1_/*n* with *Z*′ = 1 (Schmidt *et al.*, 2011 ▸) and afterwards confirmed by X-ray single-crystal diffraction (Marshall *et al.*, 2015 ▸). Barbituric acid is a completely planar, rigid, small organic compound. The molecules in the crystal exhibit a 3D hydrogen-bond network, leading to zigzag chains. For the purposes of this research, the structure published by Schmidt *et al.* (CSD reference code: IYAQOP01) was transformed to the space group 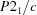, the standard space-group setting, and used as the reference structure (Fig. 4 ▸).

### 4.2. Modified structural models of barbituric acid   

Four series of 20 trial structural models, each with a modified lattice parameter *a*, *b*, *c* or β, were derived from the published structure (models A1–A20, B1–B20, C1–C20, Beta1–Beta20). They were generated by incrementally adding or subtracting 0.1 Å or 0.5°, respectively, to/from the corresponding lattice parameter, using the structure manipulation features of *FIDEL*. Consequently, each of these generated trial structural models exhibits one lattice parameter either larger or smaller than the published structural model and the molecular packing is generally correct. The molecules in the generated structures exhibit the correct molecular orientation, but the packing motif is enlarged or compressed in one dimension due to the deviation imposed.

Another 15 trial structural models were derived from the published structural model by deviating the molecular position (models P1–P5), the molecular orientation (models O1–O5) or both (models PO1–PO5) while the lattice parameters were kept at their correct values. Each of these structures exhibits a slightly different packing arrangement of the molecules.

One trial structure (model W) was modified very strongly by changing all the aforementioned parameters; it thus shares little more than the molecular geometry and the space group with the published structure.

Table 1 ▸ provides an overview of the structural changes of the trial structures compared with the correct structure (model R). The four sets of models that deviate in only one lattice parameter are each represented in Table 1 ▸ by five of the 20 generated models. Complete lists of all models appear in the supporting information (Tables S1–S4).

Furthermore, a structural model was created in which barbituric acid molecules are replaced by benzene molecules. The lattice parameters, molecular position and orientation were kept at the same values as in the correct structural model (R) of barbituric acid. Starting from this model, a series of 20 trial structural models were generated by incrementally adding 0.1 Å to the lattice parameter *b*, corresponding to the models B1–B20 derived from the published structure.

**Figure 5:**
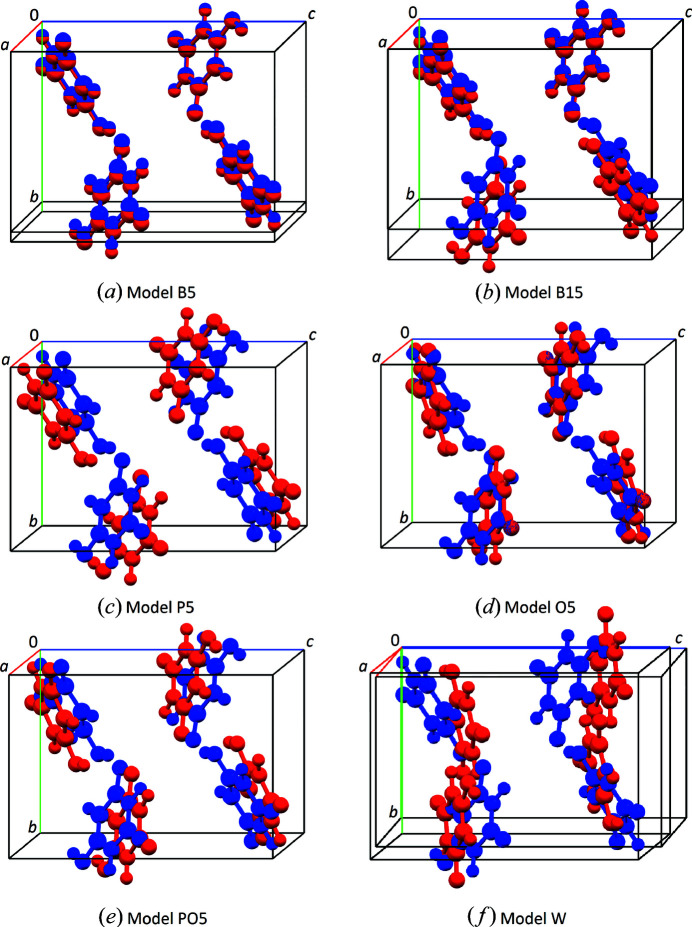
A comparison of selected trial structural models (red) with the published structure (blue).

None of the trial structures exhibits an overlap of molecules. The visual comparison of six selected trial structures with the correct structure is shown in Fig. 5 ▸.

## 5. Results and discussion   

For this research, 96 trial structures were generated, which differ from the correct, published structure (R) of polymorph IV of barbituric acid in at least one structural parameter. For each trial structure, as well as for the correct one, the PDF was calculated and compared with the experimental PDF visually and by means of 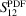, 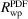 and 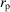.

**Figure 6:**
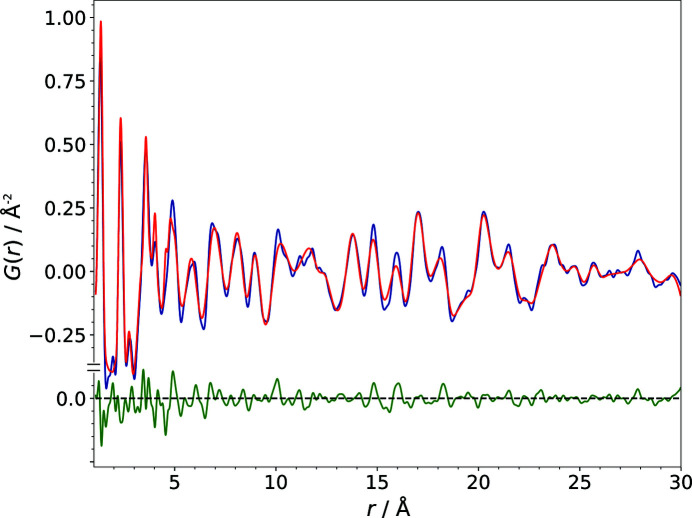
A comparison of the PDF calculated from the correct structure (model R) of barbituric acid, polymorph IV (red), with the experimental PDF (blue). The difference curve is shown in green.

The correctness of the PDF calculation from a structural model was verified by the calculation of the PDF of the published structure (R): visual comparison of the experimental and the calculated PDFs (Fig. 6 ▸), as well as the corresponding difference curve and the 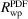 of 21.5% (which is a very good value for a PDF), proves the suitability of the procedure used to calculate the PDF from a structural model. The similarity value 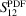 of the calculated PDF of the structure R and the experimental PDF is 0.9990, which means that the patterns are close to identical. Consequently, the correctness of the similarity measure calculation was also validated using the published structure.

### 5.1. Effects of structural changes on the PDF and the comparison measures   

In order to demonstrate the effect of a small or moderate change in one of the lattice parameters on the PDF, sets of 20 trial structural models per lattice parameter were investigated. Additionally, structural models with different molecular positions and orientations were investigated, as well as a structural model (W) that differs very much from the correct structure.

An overview of the 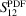, 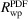 and 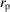 values from the comparison of the calculated PDFs of the trial structures with the experimental PDF is given in Table 2 ▸. The structural parameters of these trial structures are listed in Table 1 ▸. The deviation of the trial structures from the published one was quantified by the root mean square Cartesian displacement value (RMSCD) (van de Streek & Neumann, 2010 ▸) of all non-hydrogen atoms. The trial structures exhibited deviations from the published structure with RMSCD values of up to ∼2 Å. Even the smallest changes to the structural model with RMSCD values of 0.02–0.04 Å caused the 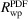 to grow from 21.5% to about 25%. Starting with still moderately deviating trial structures exhibiting RMSCD values above 0.5 Å, the 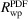 values increase from 75% to maximum values approaching 100% and reach 110% for the strongly deviating model W (RMSCD = 1.94 Å). The similarity measure 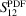
(*l* = 0.5 Å) decreases from 0.9990 for the correct structure to 0.8947 for the strongly deviating model W, while the Pearson correlation coefficient 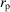 decreases from 0.9769 to 0.4242.

The following detailed discussion of the results focuses on the example of the set of trial structures (models B1–B20) that are generated by comparatively small or moderate deviations of the lattice parameter *b* and do not exceed an RMSCD of 0.45 Å.

**Figure 7:**
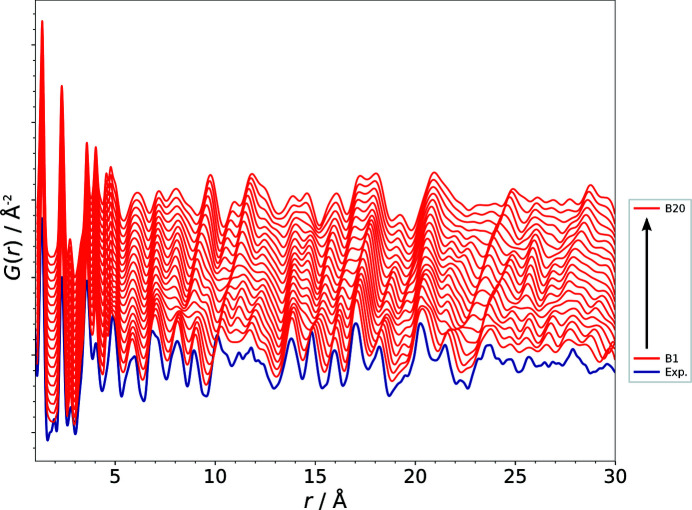
The alteration of the PDF due to the gradual modification of the lattice parameter *b* in the trial structural models B1–B20 (red). The experimental PDF of barbituric acid, polymorph IV, is shown in blue.

Fig. 7 ▸ illustrates how the PDF of a structural model changes with the gradual modification of a single parameter using the example of the lattice parameter *b* (models B1–B20). The *b* value (8.9153 Å) of the correct structure was incremented in steps of 0.1 Å up to a difference of 2 Å (∼22.4%) with respect to the lattice parameter of the published structure, corresponding to model B20 (see Table 1 ▸). Fig. 7 ▸ shows how even a moderate modification of a structural model evolves into rather large alterations in the PDF. A careful visual comparison of the calculated and experimental PDFs, however, reveals the significant similarity of the curves. It becomes apparent that the majority of the signals in the PDFs of the modified models are only shifted to slightly differing atom–atom distances. Of course the intramolecular distances, within a range of about 1–6 Å for barbituric acid, do not contribute to the changes in the calculated PDFs, as we are dealing with a rigid molecule of an unchanged geometry. With the deviation of a lattice parameter the contribution of each atom–atom pair to the PDF changes according to the component of the atom–atom vector in the direction of one lattice vector. Furthermore, with the modification of a lattice parameter the absolute values of the signal shifts increase proportionally to multiples of the length of the lattice vector with increasing atom–atom distances *r*.

Regrettably, the visual comparison of the calculated PDFs with the experimental PDF becomes tedious or even unfeasible if a large number of structural models have to be evaluated and rated against each other. Furthermore, it is obviously impossible to use visual comparison as the cost function in the fitting of a structural model to the experimental data.

### 5.2. *S*_12_^PDF^ and Pearson’s *r*   


**Figure 8:**
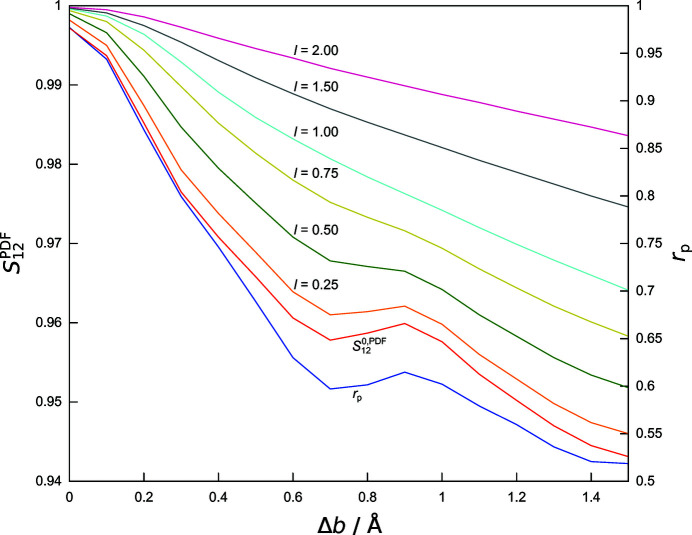
Responses of similarity measures to the alteration of the PDF due to the gradual modification of the lattice parameter *a* in the correct structure (R) and the trial structural models A1–A15. Pearson’s *r* is shown in blue, 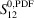 in red and similarity values 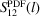 as indicated. Values of *l* are given in Å (see also Table S1).

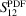
 and 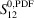 have also been compared with Pearson’s correlation coefficient 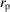 as an alternative measure of the agreement of the calculated PDFs of the trial structures with the experimental data (see Table S5 in the supporting information for all 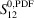 values of the structural models listed in Table 1 ▸). Fig. 8 ▸ demonstrates the close correspondence of 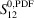 and 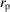 in the example of the gradual modification of the lattice parameter *a* (models A1–A20) starting from the correct structure (R). Furthermore, the figure highlights the effect of the increasing values of the neighbouring range parameter *l* (up to 2 Å) when going beyond a pointwise comparison by computation of 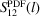. 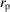 and 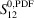 both exhibit a local minimum at about 0.7 Å above the *a* value (4.8346 Å) of the correct structure. This local minimum gradually disappears with the progressive inclusion of neighbouring data points in the comparison by broadening of the weighting function 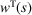 [equation (7)]. The similarity 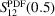 used in all the other investigations made here is already a monotonically increasing function of the lattice parameter when trial structures are approaching *a* from as far as 1.5 Å above the correct value.

### 5.3. *S*_12_^PDF^ and *R*_wp_^PDF^   

**Figure 9:**
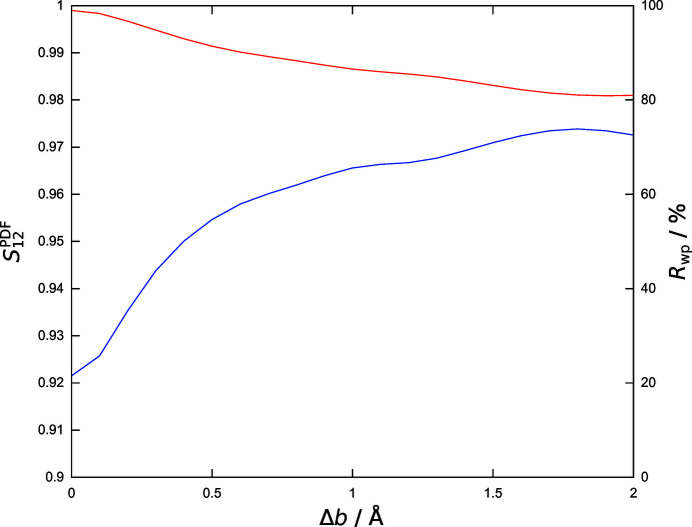
The impact of the change of the lattice parameter *b* (models R, B1–B20) on the 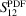 (red) and 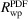 (blue) values.

Fig. 9 ▸ shows the behaviour of 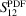 and 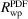 with respect to the increase of the lattice parameter *b*, corresponding to the gradual change of the calculated PDFs of the trial structures B1–B20 depicted in Fig. 7 ▸. While the change in the structural model causes a rapid increase of the 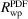 values, the decrease of the similarity measure 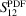 is adequately moderate and, even more importantly, it develops nearly linearly with the modification of the lattice parameter. For the modification of the lattice parameters *a*, *c* and β the behaviour of 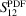 is quite similar to that shown here for the parameter *b* (see Tables S1, S2, S3 and S4 for all results). This simple example of one structural parameter changing up to ∼22.4% with respect to the correct structure highlights the drawbacks of the use of 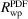 and of the comparison based on pointwise differences in general. While 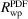 suits the tracking of small changes very well (up to a deviation of *b* by ∼0.5 Å), it cannot adequately reflect the considerable agreement of the structural model with the experimental data if the modification exceeds a certain, although still moderate, magnitude. In the case of the example of the impact of the modification of a single lattice parameter as shown in Fig. 7 ▸, 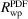 becomes largely unsuitable on modification of *b* by more than ∼1.0 Å.

Beyond a rather narrow parameter hyperspace close to the correct structural description, this behaviour of 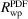 represents a major challenge to the setting of a meaningful threshold value or to the use of 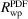 or related measures as the cost function in the fitting of a structural model to the experimental data. Hence, the use of the similarity measure 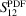 for a PDF comparison provides a valuable solution to these problems. While the demonstrated behaviour of the similarity measure 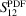 already makes it a favourable tool, it becomes even more suitable in the light of the possibility of adapting 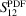 to the characteristics of the problem or to the application scenario by adjustment of the parameter *l* [equation (7)], which controls the ‘neighbourhood awareness’ of the comparison.

**Figure 10:**
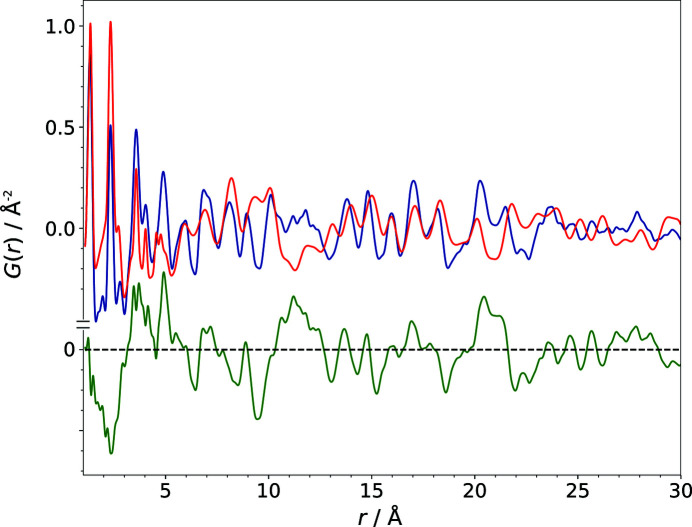
The comparison of the calculated PDF of the structural model P5 (red) with the experimental PDF (blue) of barbituric acid, polymorph IV. The difference curve is shown in green.

The structural models P1–P5, O1–O5 and PO1–PO5 were generated by changes in the position and/or orientation of the molecule, while the lattice parameters were those of the published structure (see Table 1 ▸). The 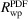 values of these models were all above 50%, with the highest 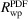 of 98.1% found for model P5 (Table 2 ▸). The impact of the modification of 0.08 in each fractional coordinate imposed in model P5 leads to a difference curve that showed many strong discrepancies (Fig. 10 ▸). Hence, 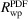 was completely incapable of reflecting the fact that the molecule and the lattice parameters of this trial structure were the same as in the correct structure. Again, by comparison based on pointwise differences of the calculated and the experimental PDFs, most of these structural models would be considered as inadequate descriptions of the experimental data. However, one look at the comparison of the models P5, O5 and PO5 with the correct structure shows that the structures were still roughly congruent [Figs. 5 ▸(*c*)–5 ▸(*e*)]. The 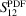 values in contrast adequately express the considerable congruence of these models with the published structure, which becomes evident by visual comparison of the crystal structures as well as by looking at the corresponding RMSCD values listed in Table 2 ▸.

The general logic of *R* values is that they quantify the residual percentage of the observation that is not explained by the model. A value of 100% corresponds to a ‘model’ that explains nothing at all, *e.g.* the simple reference case where the difference curve is identical to the experimental curve.

**Figure 11:**
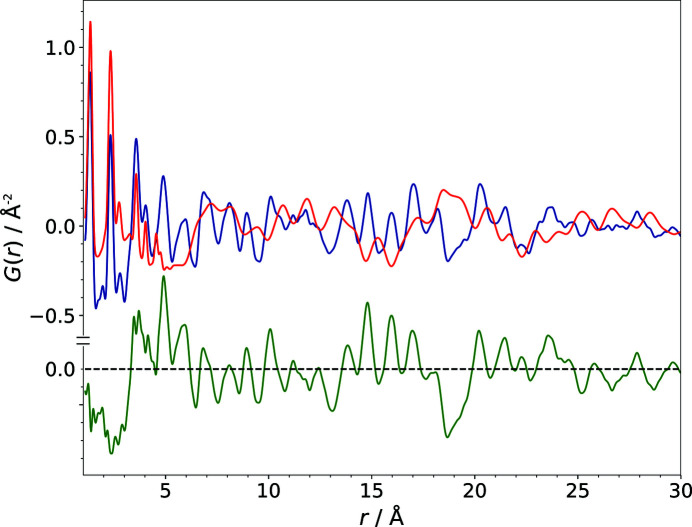
The comparison of the calculated PDF of the structural model W (red) with the experimental PDF (blue) of barbituric acid, polymorph IV. The difference curve is shown in green.

The comparison of the calculated PDF of the strongly modified structural model W with the experimental PDF is shown in Fig. 11 ▸. The lowest 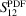 (0.8947) of all trial structures was calculated for this model, which shares not much more than the space group and the molecular geometry with the published structure of polymorph IV of barbituric acid [see Fig. 5 ▸(*f*)].

The 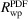 value of 109.8% resulting from the comparison of model W with the experimental data is already far beyond the ranges of the reasonable application of *R* values. This value above 100% expresses that the model not only failed to explain the observed data, but even aggravated the situation by giving false explanations. Note that the design of the weighted-pattern *R* values is directed at the evaluation of model results versus experimental data, which cannot be negative, and thus in cases like the analysis of powder patterns the possible range of 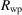 is strictly limited to 0–100%. Inspection of the enormous amplitudes of the difference curve in Fig. 11 ▸ elucidates very well why the 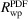 is above 100% and why the comparison based on pointwise differences fails in the recognition of the still considerable similarities of the structural model and its calculated PDF with the correct structure and the experimental PDF.

### 5.4. Absolute values of *S*_12_^PDF^ and the effects of intra- and intermolecular distances   

In contrast to the comparison of powder patterns by calculation of 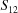 (*e.g.* Habermehl *et al.*, 2014 ▸) the values of 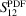 are usually very high. Basically, this reflects the fact that all radial distributions of condensed matter exhibit a significant degree of similarity. The PDF as described in equation (1) is defined as a difference with respect to the average atomic number density 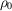. The definition of 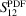 based on the transformation of 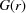 according to equation (9) implies that 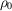 is the same for the two PDFs compared, which is a reasonable assumption if both PDFs represent the same compound. But also, beyond that, it is still an arguable assumption for any condensed organic phase.

Intramolecular atom–atom distances of organic compounds exhibit a high degree of similarity between different compounds, due to *e.g.* typical bond lengths and ring or chain geometries. In particular the PDFs of the same molecule inevitably have a lot in common, and the larger and more rigid the molecules, the higher the degree of similarity of the PDFs. Typical intermolecular distances are another source of similarity, *e.g.* considering π–π stacking, hydrogen-bond networks and the limited range of van der Waals distances in solids. Furthermore, most of these exact or typical similarities contribute to the PDF in the low-*r* region where the PDF exhibits the highest amplitudes. However, the generally high values of 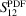 should not be seen as a drawback, because the essential advantages of this similarity measure for PDFs lie in the way it reflects structural changes, as explained in the discussion of Fig. 9 ▸.

**Figure 12:**
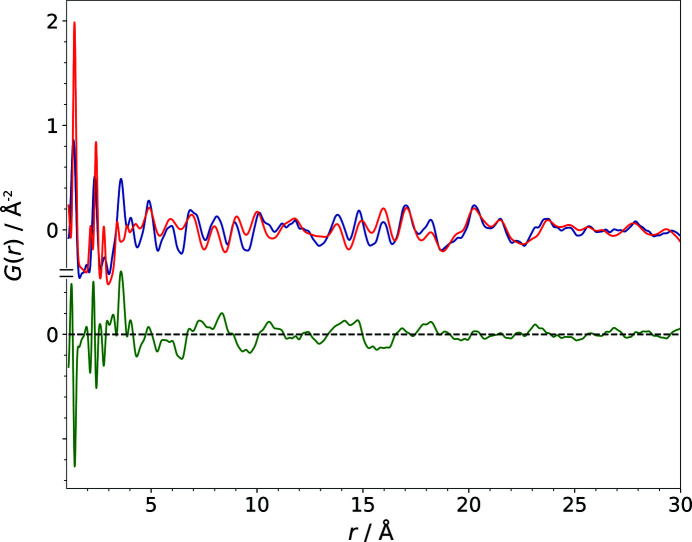
The comparison of the calculated PDF (red) of the structural model in which benzene replaced the barbituric acid molecules of the correct structure R with the experimental PDF (blue) of barbituric acid, polymorph IV. The difference curve is shown in green.

**Figure 13:**
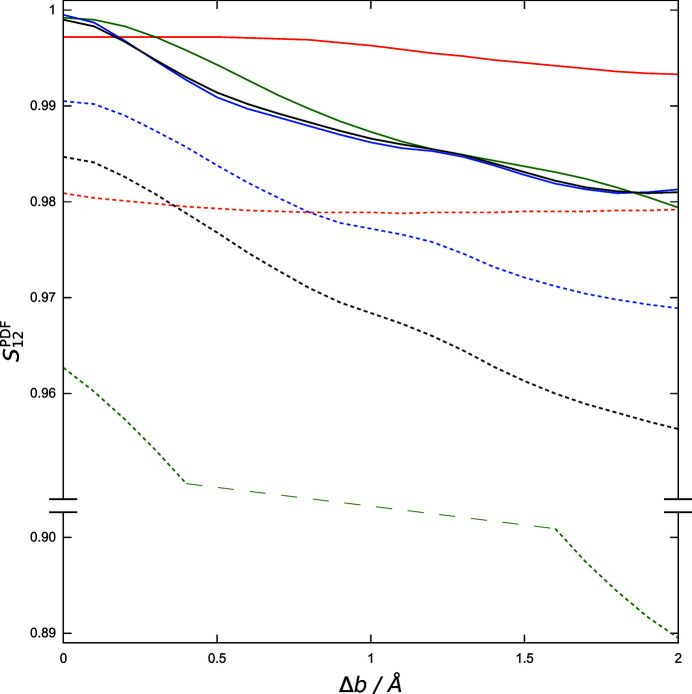
The impact of the change of the lattice parameter *b* (models R, B1–B20) on the 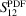 values computed for the full range of the PDF (1.1–30 Å, black line, see also Fig. 7 ▸) and for the ranges of intramolecular distances (1.1–3.3 Å, red), intermolecular distances (6–30 Å, blue) and the overlap region (3.3–6 Å, green). Solid lines represent structural models of barbituric acid and dashed lines the corresponding models where barbituric acid was replaced by benzene.

The specific response of 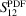 to intramolecular and intermolecular distances has been investigated by computation of the similarities for three distinct sub-ranges of the PDF and evaluation of structural models where barbituric acid was replaced with benzene. Fig. 12 ▸ clearly shows the substantial alteration of the simulated PDF induced by switching from barbituric acid to benzene, viewed in contrast to the corresponding comparison of the same experimental data with the calculated PDF of the correct structure (Fig. 6 ▸). Fig. 13 ▸ demonstrates how the similarity values calculated for different regions of the PDF respond to the replacement of the mol­ecule and to structural changes using the example of the gradual modification of the lattice parameter *b*.

The 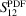 values of the structural models of barbituric acid for the full range of the PDF (see Fig. 9 ▸) are primarily dominated by differences in intermolecular distances (starting at about 6 Å). The structural similarity of corresponding trial structures containing different molecules is still reflected by their 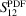 values for the region of intermolecular distances.

The similarities in the intramolecular region of the PDF (up to 3.3 Å) are practically invariant for all models containing the same molecule. The slight drop in the similarity of trial structures of barbituric acid with *b* differing from the correct structure by more than about 1 Å reveals some short intermolecular distances in the structures, which were generated excluding explicit overlap of molecules but not required to be chemically reasonable. Consequently, the replacement of the barbituric acid molecule in the structural models by the slightly smaller 6-ring molecule of benzene results in practically undisturbed constant intramolecular similarities, besides the decrease compared with corresponding values for barbituric acid. The simulated PDF of a correct structural model yields slightly smaller similarities in the low-*r* range compared with those computed over a wider range, which may at least partially be attributed to the high amplitudes and the limited reliability of the experimental PDF in that region.

The highest variability and sensitivity in the response of 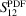 values to structural changes were found in the region 3.3–6 Å, where contributions of intra- and intermolecular distances overlap (see Table S5). Correspondingly the impact of replacing barbituric acid by benzene on the similarity values of the simulated PDFs is most pronounced in that region (Fig. 13 ▸).

## 6. Conclusion   

In this work a new method for the comparison of pair distribution functions, using a similarity measure based on cross-correlation functions, was introduced. The potential of the proposed similarity measure 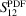 was evaluated with the example of polymorph IV of barbituric acid, focusing on the comparison of the calculated PDFs of modified trial structures with the experimental PDF obtained from a suitable powder diffraction measurement.

It was shown that the PDF is very sensitive to changes in the local structure and that the comparison of PDFs based on pointwise differences (*e.g. R* values and difference curves) tends to fail in adequately detecting and expressing a rough congruence of the PDFs. The investigated 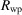 was proven to be suitable for the quantification of the match between the investigated structures and the experimental data if the structural models are already very close to the correct one. The *R* values for modified structural models, however, become very large and insensitive in the case of even moderate modifications with respect to the correct structure. This often leads to the wrong assumption that the investigated PDFs and the corresponding structures are in no agreement at all, even in the case of comparatively moderate deviations of a basically correct model.

The alternative approach to the comparison of simulated and experimental PDFs by the calculation of the similarity value 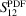 has proved to be feasible, and rough matches of structural models could be detected by the measure rather well. Moderate modifications in a structural model used for the calculation of the PDF did not cause a crucial variance in the similarity value, but were reflected in a gradual response of the 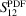 value, which favours the setting of threshold values or the fitting of structural models to an experimental PDF. Hence, the measure 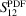 can be a meaningful and useful tool for structural investigations in which a large number of structures have to be evaluated via PDF comparison and prospective structural models need to be identified for further investigation.

The deployment of cross-correlation functions in 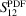 introduces a well defined and scalable ‘neighbourhood awareness’ by including a certain neighbouring range in the correlation of the values of the two PDFs at corresponding atom-pair distances. Thereby the response of 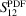 to changes in structural models can easily be tuned in order to best suit specific tasks and problems. The limit of 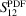 on narrowing the neighbouring range down to zero corresponds to the Pearson correlation coefficient 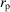, a similar but less powerful measure with which 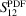 was also compared.

The specific response of 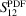 to contributions of intra­molecular and intermolecular distances to the PDF has also been investigated. 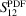 values for the full range of the experimental PDF were primarily dominated by the alterations in exclusively intermolecular distances. The largest variability of 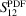 was found in the sub-range similarity values corresponding to the region of overlapping inter- and intramolecular contributions to the PDF. Range-specific similarities for the exclusively intramolecular region of the PDF were not affected by reasonable structural changes, but did allow the differentiation of similar molecules.

The characteristics of 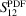 favour its use as a valuable and general tool for various purposes:

(i) The effective screening and ranking of large numbers of structural models (*e.g.* the results of a structure prediction) against an experimental PDF.

(ii) The comparison and fitting of structural models to an experimental PDF during a structure solution by a fit to the PDF (Schlesinger *et al.*, 2021 ▸).

(iii) The detection of dominant or preferred local structure motifs by the comparison of structural models with the experimental PDF of apparently amorphous samples [see Billinge *et al.* (2010 ▸)].

(iv) The comparison and clustering of structural models according to their local structure.

(v) The comparison of experimental PDFs with each other, in particular if they come from different sources or have been obtained from measurements under different conditions.

(vi) The clustering of large numbers of experimental PDFs with respect to the local structure of the investigated materials.

The proposed similarity measure 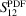 addresses the general problem confronting local structure analyses based on the PDF, which results from the increased variability and complexity of local structural arrangements. Therefore, this novel approach may open the way to considerable advances in the further exploration of its application in the investigation of the local structure of nanocrystalline, amorphous or complex nanostructured materials.

## Supplementary Material

Supporting information file. DOI: 10.1107/S1600576721001722/kc5119sup1.pdf

## Figures and Tables

**Table 1:** Structural parameters of the correct structure (R) and 36 trial structural models of barbituric acid in space group *P*2_1_/*c* with *Z*′ = 1 The molecules were shifted or rotated with respect to the lattice vectors according to the values of \Delta m and \Delta\varphi, which signify the change relative to the correct structure applied to each of the three components of the fractional position (\Delta m = \Delta m_{x} = \Delta m_{y} = \Delta m_{z}) or the molecular orientation (\Delta\varphi = \Delta\varphi_{x} = \Delta\varphi_{y} = \Delta\varphi_{z}).

Structural model	*a* (Å)	*b* (Å)	*c* (Å)	β (°)	\Delta m	\Delta\varphi (°)
R	4.8346	8.9153	12.4192	107.729	0.00	0.0
A1	4.9346	8.9153	12.4192	107.729	0.00	0.0
A5	5.3346	8.9153	12.4192	107.729	0.00	0.0
A10	5.8346	8.9153	12.4192	107.729	0.00	0.0
A15	6.3346	8.9153	12.4192	107.729	0.00	0.0
A20	6.8346	8.9153	12.4192	107.729	0.00	0.0
B1	4.8346	9.0153	12.4192	107.729	0.00	0.0
B5	4.8346	9.4153	12.4192	107.729	0.00	0.0
B10	4.8346	9.9153	12.4192	107.729	0.00	0.0
B15	4.8346	10.4153	12.4192	107.729	0.00	0.0
B20	4.8346	10.9153	12.4192	107.729	0.00	0.0
C1	4.8346	8.9153	12.3192	107.729	0.00	0.0
C5	4.8346	8.9153	11.9192	107.729	0.00	0.0
C10	4.8346	8.9153	11.4192	107.729	0.00	0.0
C15	4.8346	8.9153	10.9192	107.729	0.00	0.0
C20	4.8346	8.9153	10.4192	107.729	0.00	0.0
Beta1	4.8346	8.9153	12.4192	108.229	0.00	0.0
Beta5	4.8346	8.9153	12.4192	110.229	0.00	0.0
Beta10	4.8346	8.9153	12.4192	112.729	0.00	0.0
Beta15	4.8346	8.9153	12.4192	115.229	0.00	0.0
Beta20	4.8346	8.9153	12.4192	117.729	0.00	0.0
P1	4.8346	8.9153	12.4192	107.729	−0.03	0.0
P2	4.8346	8.9153	12.4192	107.729	−0.04	0.0
P3	4.8346	8.9153	12.4192	107.729	0.04	0.0
P4	4.8346	8.9153	12.4192	107.729	0.06	0.0
P5	4.8346	8.9153	12.4192	107.729	0.08	0.0
O1	4.8346	8.9153	12.4192	107.729	0.00	−10.0
O2	4.8346	8.9153	12.4192	107.729	0.00	−15.0
O3	4.8346	8.9153	12.4192	107.729	0.00	9.0
O4	4.8346	8.9153	12.4192	107.729	0.00	10.0
O5	4.8346	8.9153	12.4192	107.729	0.00	11.0
PO1	4.8346	8.9153	12.4192	107.729	0.02	2.0
PO2	4.8346	8.9153	12.4192	107.729	0.03	3.0
PO3	4.8346	8.9153	12.4192	107.729	0.04	4.0
PO4	4.8346	8.9153	12.4192	107.729	0.04	5.0
PO5	4.8346	8.9153	12.4192	107.729	0.04	6.0
W	7.8346	11.9153	13.4192	117.729	0.08	10.0

**Table 2:** RMSCD, R_{\rm wp}^{{\rm PDF}}, S_{12}^{{\rm PDF}} and r_{\rm p} values for the correct structure (R) and 36 trial structural models (see Table 1 ▸) of barbituric acid in space group *P*2_1_/*c* The S_{12}^{{\rm PDF}} values listed were calculated with a neighbouring range parameter *l* of 0.5 Å [see equation (7)].

Structural model	RMSCD (Å)	R_{\rm wp}^{{\rm PDF}} (%)	S_{12}^{{\rm PDF}}	r_{\rm p}
R	0.000	21.486	0.9990	0.9769
A1	0.071	33.489	0.9966	0.9438
A5	0.362	77.257	0.9751	0.6887
A10	0.743	86.124	0.9642	0.6012
A15	1.140	94.476	0.9518	0.5186
A20	1.550	92.932	0.9558	0.5420
B1	0.021	25.738	0.9983	0.9666
B5	0.109	54.650	0.9914	0.8425
B10	0.218	65.591	0.9866	0.7742
B15	0.329	70.963	0.9831	0.7373
B20	0.441	72.561	0.9810	0.7202
C01	0.035	24.552	0.9984	0.9697
C05	0.176	47.957	0.9921	0.8823
C10	0.350	68.473	0.9818	0.7568
C15	0.523	76.854	0.9770	0.6841
C20	0.695	78.779	0.9759	0.6707
Beta1	0.028	24.464	0.9986	0.9699
Beta5	0.142	47.097	0.9927	0.8848
Beta10	0.284	64.061	0.9851	0.7837
Beta15	0.425	71.010	0.9813	0.7460
Beta20	0.564	80.066	0.9758	0.6886
P1	0.240	55.200	0.9882	0.8355
P2	0.320	59.309	0.9841	0.8102
P3	0.533	72.550	0.9796	0.7045
P4	0.627	85.219	0.9620	0.6117
P5	0.724	98.093	0.9428	0.5244
O1	0.504	58.540	0.9881	0.8151
O2	0.730	64.151	0.9848	0.7769
O3	0.495	72.649	0.9773	0.7147
O4	0.552	77.677	0.9725	0.6775
O5	0.610	82.078	0.9671	0.6429
PO1	0.217	52.412	0.9905	0.8528
PO2	0.330	71.259	0.9804	0.7153
PO3	0.445	86.359	0.9644	0.5759
PO4	0.483	89.400	0.9544	0.5456
PO5	0.525	92.599	0.9331	0.5177
W	1.940	109.842	0.8947	0.4242
