# Supplementary material for: Comparison and evaluation of pair distribution functions, using a similarity measure based on cross-correlation functions
Source: J Appl Crystallogr. 2021 Mar 31;54(Pt 2):612–23. doi: 10.1107/S1600576721001722 (PMC8056768; doi:10.1107/S1600576721001722)
Supplement: Supplementary file 1 [file j-54-00612-sup1.pdf]

JOURNAL OF APPLIED CRYSTALLOGRAPHY

Volume 54 (2021)

Supporting information for article:

Comparison and evaluation of pair distribution functions, using a similarity measure based on cross-correlation functions

Stefan Habermehl, Carina Schlesinger and Dragica Prill

Journal of Applied Crystallography

ISSN 1600-5767

Comparison and evaluation of pair distribution functions, using a similarity measure based on cross-correlation functions - Supporting information

Stefan Habermehl, Carina Schlesinger and Dragica Prill *

Institute of Inorganic and Analytical Chemistry, Goethe University, Max-von-Laue-Str. 7, 60438 Frankfurt am Main, Germany. Correspondence e-mail: prill@chemie.uni-frankfurt.de

See: doi:10.1107/S1600576721001722

Tables S1, S2, S3 and S4 provide an overview of structural changes as well as resulting S_{12}^{PDF}, RMSCD and R_{wp}^{PDF} values for the four sets of 20 trial structures that deviate in only one lattice parameter.

Table S5 lists the similarity values $S_{12}^{\mathrm{PDF}}(l\text{=}0.5\ \text{Å})$ computed for different ranges of r as well as the $S_{12}^{0,\mathrm{PDF}}$ (for the full range) of all trial structures of table 1 of the publication.

Table S1

Structural parameters and corresponding RMSCD, S_{12}^{PDF}, R_{wp}^{PDF}, $S_{12}^{0,\mathrm{PDF}}$ and r_p values for the correct structure (R) and 20 trial structural models (A1-A20) of barbituric acid in space group $P\,2_1/c$ that exhibit the modification of the lattice parameter a.

Structural model	a / Å	b / Å	c / Å	β / °	RMSCD / Å	R_{wp}^{PDF} / %	S_{12}^{PDF}	$S_{12}^{0,\mathrm{PDF}}$	r_p
R	4.8346	8.9153	12.4192	107.729	0.000	21.486	0.9990	0.9972	0.9769
A1	4.9346	8.9153	12.4192	107.729	0.071	33.489	0.9966	0.9938	0.9438
A2	5.0346	8.9153	12.4192	107.729	0.143	50.983	0.9911	0.9854	0.8696
A3	5.1346	8.9153	12.4192	107.729	0.215	63.060	0.9847	0.9768	0.7990
A4	5.2346	8.9153	12.4192	107.729	0.288	70.297	0.9795	0.9711	0.7460
A5	5.3346	8.9153	12.4192	107.729	0.362	77.257	0.9751	0.9663	0.6887
A6	5.4346	8.9153	12.4192	107.729	0.437	84.035	0.9708	0.9611	0.6291
A7	5.5346	8.9153	12.4192	107.729	0.513	87.225	0.9678	0.9584	0.5966
A8	5.6346	8.9153	12.4192	107.729	0.589	86.203	0.9671	0.9592	0.6003
A9	5.7346	8.9153	12.4192	107.729	0.666	84.494	0.9665	0.9604	0.6135
A10	5.8346	8.9153	12.4192	107.729	0.743	86.124	0.9642	0.9582	0.6012
A11	5.9346	8.9153	12.4192	107.729	0.821	89.113	0.9610	0.9542	0.5785
A12	6.0346	8.9153	12.4192	107.729	0.900	91.152	0.9583	0.9509	0.5593
A13	6.1346	8.9153	12.4192	107.729	0.979	93.100	0.9556	0.9477	0.5361
A14	6.2346	8.9153	12.4192	107.729	1.060	94.340	0.9534	0.9451	0.5206
A15	6.3346	8.9153	12.4192	107.729	1.140	94.476	0.9518	0.9437	0.5186
A16	6.4346	8.9153	12.4192	107.729	1.220	94.027	0.9505	0.9429	0.5246
A17	6.5346	8.9153	12.4192	107.729	1.300	93.397	0.9496	0.9424	0.5324
A18	6.6346	8.9153	12.4192	107.729	1.380	93.541	0.9512	0.9442	0.5308
A19	6.7346	8.9153	12.4192	107.729	1.470	93.812	0.9539	0.9471	0.5286
A20	6.8346	8.9153	12.4192	107.729	1.550	92.932	0.9558	0.9495	0.5420

Table S2

Structural parameters and corresponding RMSCD, S_{12}^{PDF} and R_{wp}^{PDF} values for the correct structure (R) and 20 trial structural models (B1-B20) of barbituric acid in space group $P\,2_1/c$ that exhibit the modification of the lattice parameter b.

Structural model	a / Å	b / Å	c / Å	β / °	RMSCD / Å	R_{wp}^{PDF} / %	S_{12}^{PDF}
R	4.8346	8.9153	12.4192	107.729	0.000	21.486	0.998
B1	4.8346	9.0153	12.4192	107.729	0.021	25.737	0.998
B2	4.8346	9.1153	12.4192	107.729	0.043	35.301	0.996
B3	4.8346	9.2153	12.4192	107.729	0.065	43.798	0.994
B4	4.8346	9.3153	12.4192	107.729	0.086	50.083	0.992
B5	4.8346	9.4153	12.4192	107.729	0.109	54.650	0.991
B6	4.8346	9.5153	12.4192	107.729	0.130	57.923	0.990
B7	4.8346	9.6153	12.4192	107.729	0.152	60.102	0.989
B8	4.8346	9.7153	12.4192	107.729	0.174	61.950	0.988
B9	4.8346	9.8153	12.4192	107.729	0.196	63.938	0.987
B10	4.8346	9.9153	12.4192	107.729	0.218	65.590	0.986
B11	4.8346	10.0153	12.4192	107.729	0.240	66.340	0.985
B12	4.8346	10.1153	12.4192	107.729	0.262	66.707	0.985
B13	4.8346	10.2153	12.4192	107.729	0.284	67.660	0.984
B14	4.8346	10.3153	12.4192	107.729	0.307	69.262	0.984
B15	4.8346	10.4153	12.4192	107.729	0.329	70.962	0.983
B16	4.8346	10.5153	12.4192	107.729	0.351	72.422	0.982
B17	4.8346	10.6153	12.4192	107.729	0.373	73.460	0.981
B18	4.8346	10.7153	12.4192	107.729	0.396	73.857	0.981
B19	4.8346	10.8153	12.4192	107.729	0.418	73.447	0.980
B20	4.8346	10.9153	12.4192	107.729	0.441	72.560	0.980

Table S3

Structural parameters and corresponding RMSCD, S_{12}^{PDF} and R_{wp}^{PDF} values for the correct structure (R) and 20 trial structural models (C1-C20) of barbituric acid in space group $P\,2_1/c$ that exhibit the modification of the lattice parameter c.

Structural model	a / Å	b / Å	c / Å	β / °	RMSCD / Å	R_{wp}^{PDF} / %	S_{12}^{PDF}
R	4.8346	8.9153	12.4192	107.729	0.000	21.486	0.998
C1	4.8346	8.9153	12.3192	107.729	0.035	24.552	0.998
C2	4.8346	8.9153	12.2192	107.729	0.070	30.395	0.997
C3	4.8346	8.9153	12.1192	107.729	0.105	36.735	0.995
C4	4.8346	8.9153	12.0192	107.729	0.141	42.680	0.994
C5	4.8346	8.9153	11.9192	107.729	0.176	47.957	0.992
C6	4.8346	8.9153	11.8192	107.729	0.211	52.683	0.989
C7	4.8346	8.9153	11.7192	107.729	0.246	57.067	0.987
C8	4.8346	8.9153	11.6192	107.729	0.280	61.213	0.985
C9	4.8346	8.9153	11.5192	107.729	0.315	65.089	0.983
C10	4.8346	8.9153	11.4192	107.729	0.350	68.473	0.981
C11	4.8346	8.9153	11.3192	107.729	0.385	70.994	0.980
C12	4.8346	8.9153	11.2192	107.729	0.420	72.664	0.979
C13	4.8346	8.9153	11.1192	107.729	0.454	73.947	0.978
C14	4.8346	8.9153	11.0192	107.729	0.489	75.306	0.977
C15	4.8346	8.9153	10.9192	107.729	0.523	76.854	0.977
C16	4.8346	8.9153	10.8192	107.729	0.558	78.292	0.976
C17	4.8346	8.9153	10.7192	107.729	0.592	79.237	0.975
C18	4.8346	8.9153	10.6192	107.729	0.627	79.527	0.975
C19	4.8346	8.9153	10.4192	107.729	0.661	79.318	0.975
C20	4.8346	8.9153	10.4192	107.729	0.695	78.778	0.975

Table S4

Structural parameters and corresponding RMSCD, S_{12}^{PDF} and R_{wp}^{PDF} values for the correct structure (R) and 20 trial structural models (Beta1-Beta20) of barbituric acid in space group $P\,2_1/c$ that exhibit the modification of the lattice parameter β.

Structural model	a / Å	b / Å	c / Å	β / °	RMSCD / Å	R_{wp}^{PDF} / %	S_{12}^{PDF}
R	4.8346	8.9153	12.4192	107.729	0.000	21.486	0.998
Beta1	4.8346	8.9153	12.4192	108.229	0.028	24.464	0.998
Beta2	4.8346	8.9153	12.4192	108.729	0.057	30.162	0.997
Beta3	4.8346	8.9153	12.4192	109.229	0.085	36.468	0.996
Beta4	4.8346	8.9153	12.4192	109.729	0.114	42.180	0.994
Beta5	4.8346	8.9153	12.4192	110.229	0.142	47.096	0.992
Beta6	4.8346	8.9153	12.4192	110.729	0.171	51.354	0.990
Beta7	4.8346	8.9153	12.4192	111.229	0.199	55.162	0.989
Beta8	4.8346	8.9153	12.4192	111.729	0.227	58.563	0.987
Beta9	4.8346	8.9153	12.4192	112.229	0.256	61.533	0.986
Beta10	4.8346	8.9153	12.4192	112.729	0.284	64.060	0.985
Beta11	4.8346	8.9153	12.4192	113.229	0.312	66.146	0.984
Beta12	4.8346	8.9153	12.4192	113.729	0.340	67.783	0.983
Beta13	4.8346	8.9153	12.4192	114.229	0.368	68.993	0.982
Beta14	4.8346	8.9153	12.4192	114.729	0.397	69.972	0.981
Beta15	4.8346	8.9153	12.4192	115.229	0.425	71.009	0.981
Beta16	4.8346	8.9153	12.4192	115.729	0.452	72.296	0.980
Beta17	4.8346	8.9153	12.4192	116.229	0.480	73.920	0.979
Beta18	4.8346	8.9153	12.4192	116.729	0.508	75.888	0.978
Beta19	4.8346	8.9153	12.4192	117.229	0.523	78.039	0.977
Beta20	4.8346	8.9153	12.4192	117.729	0.564	80.066	0.975

Table S5

Comparison of the structural models of the tables 1 and 2 of the publication to the experimental PDF of barbituric acid: Similarity values $S_{12}^{\mathrm{PDF}}(l{=}0.5\ \text{Å})$ computed for different ranges of r and $S_{12}^{0,\mathrm{PDF}}$ for the full range.

Structural model		S_{12}^{PDF}	S_{12}^{PDF}	S_{12}^{PDF}	S_{12}^{PDF}	$S_{12}^{0,\mathrm{PDF}}$
	Range:	1.1-3.3 Å	3.3-6 Å	6-30 Å	1.1-30 Å	1.1-30 Å
R		0.9972	0.9992	0.9995	0.9990	0.9972
A1		0.9971	0.9992	0.9967	0.9966	0.9938
A5		0.9932	0.9719	0.9756	0.9751	0.9663
A10		0.9898	0.9253	0.9713	0.9642	0.9582
A15		0.9870	0.8786	0.9656	0.9518	0.9437
A20		0.9854	0.8697	0.9727	0.9558	0.9495
B1		0.9972	0.9990	0.9987	0.9983	0.9962
B5		0.9972	0.9943	0.9909	0.9914	0.9849
B10		0.9963	0.9873	0.9862	0.9866	0.9794
B15		0.9945	0.9837	0.9828	0.9831	0.9763
B20		0.9933	0.9794	0.9813	0.9810	0.9749
C1		0.9969	0.9992	0.9988	0.9984	0.9962
C5		0.9979	0.9986	0.9910	0.9921	0.9879
C10		0.9975	0.9976	0.9784	0.9818	0.9764
C15		0.9962	0.9953	0.9731	0.9770	0.9710
C20		0.9948	0.9929	0.9721	0.9759	0.9696
Beta1		0.9971	0.9989	0.9990	0.9986	0.9964
Beta5		0.9961	0.9966	0.9923	0.9927	0.9876
Beta10		0.9962	0.9930	0.9835	0.9851	0.9789
Beta15		0.9951	0.9934	0.9790	0.9813	0.9761
Beta20		0.9962	0.9926	0.9722	0.9758	0.9702
P1		0.9978	0.9776	0.9898	0.9882	0.9830
P2		0.9963	0.9721	0.9861	0.9841	0.9787
P3		0.9788	0.9890	0.9831	0.9796	0.9718
P4		0.9640	0.9830	0.9740	0.9620	0.9550
P5		0.9526	0.9844	0.9633	0.9428	0.9380
O1		0.9949	0.9779	0.9901	0.9881	0.9818
O2		0.9978	0.9743	0.9862	0.9848	0.9776
O3		0.9885	0.9712	0.9799	0.9773	0.9711
O4		0.9884	0.9648	0.9755	0.9725	0.9658
O5		0.9894	0.9581	0.9706	0.9671	0.9596
PO1		0.9926	0.9823	0.9912	0.9905	0.9857
PO2		0.9837	0.9957	0.9711	0.9804	0.9733
PO3		0.9813	0.9939	0.9831	0.9644	0.9548
PO4		0.9856	0.9708	0.9630	0.9544	0.9434
PO5		0.9897	0.9442	0.9458	0.9331	0.9181
W		0.9794	0.8419	0.9393	0.8947	0.8818
